# Prevalence and preventive measures of spinal musculoskeletal disorders among dentists at the University Dental Hospital of Monastir: a cross-sectional study

**DOI:** 10.11604/pamj.2025.52.40.36567

**Published:** 2025-09-25

**Authors:** Irtyah Merchaoui, Farah Chelly, Samia Machghoul, Marouan Hayouni, Imane El kharras, Mohamed Khlifa, Ines Rassas, Neila Chaari, Mohamed Adnène Henchi, Mohamed Akrout

**Affiliations:** 1 Department of Occupational Medicine, Fattouma Bourguiba University Hospital-Monastir, Monastir, Tunisia,; 2 Laboratory of Ergonomic Management of Professional Risk and Environment, Monastir, Tunisia,; 3 Department of Occupational Medicine, Sahloul University Hospital, Faculty of Medicine of Sousse, University of Sousse, Sousse, Tunisia,; 4 Department of Stomatology, University Hospital Clinic of Odontology, Monastir, Tunisia

**Keywords:** Musculoskeletal disorders, spine, dentist

## Abstract

**Introduction:**

Musculoskeletal disorders (MSDs), particularly those affecting the spine, are highly prevalent among dentists due to the prolonged static postures and repetitive movements inherent to dental practice. Understanding their prevalence and associated risk factors is crucial for implementing effective preventive strategies and ensuring the well-being and career longevity of dental professionals.

**Methods:**

this is a cross-sectional study over 2 months, conducted among dentists of the university hospital of odontology in Monastir. The data collection was carried out by means of a self-questionnaire exploring the sociodemographic and professional data of the participants. The NORDIC style questionnaire and the KARASEK questionnaire were used to explore musculoskeletal health and psychosocial constraints, respectively.

**Results:**

the final sample size of our study consisted of 260 eligible individuals, with a final participation rate of 64.23%. The typical profile of the participants was predominantly female 70.7% (n=118), right-handed 90.4% (n=151), with a mean age of 28.30 ± 6.7 years. Most were single 75.4% (n=127), non-smokers 73.7% (n=123) and engaged in physical activity 56.1% (n=93). A total of 83.8% (n=141) of participants were dental trainees. The majority of practitioners, 58.7% (n=98), were working in departments focused exclusively on medical dental practice. The median seniority was 12 months, while the average number of daily working hours was 5 ± 1.16 hours. The prevalence of MSDs of the spine, all sites combined, was 67.6% (n=113). According to the binary regression model, the determinants of cervical pain risk were: gender (OR: 0.42, 95% CI: 0.18-0.96; p=0.04); inter-patient breaks (OR: 0.24, 95% CI 0.1-0.58; p=0.002); and the concomitant presence of back pain and low back pain (OR: 3.8, 95% CI: 95 1.6-8.8; p=0.001) and (OR: 6.2, 95% CI 2.7-14.5; p=10-3) respectively. As for back pain, the risk was reduced by a factor of 0.79 in the case of frequent rotation of practitioners working in the same chair (OR: 0.79, 95% CI 0.66-0.95; p=0.01) and increased by the presence of other MSD (OR: 1.43, 95% CI: 1.1-1.87; p= 0.007); by the absence of break sessions (OR: 2.58, 95% CI 1.24-5.38; p=0.01) and by the concomitant presence of neck pain (OR: 3.34, 95% CI: 1.48-7.54; p=0.004). The risk of developing low back pain was increased by high daily hourly mass (OR: 4.9, 95% CI 2.2-11.1; p=0.001); in case of concomitant cervical pain (OR: 2.88, 95% CI: 1.3-6.2; p=0.007) and in case of a learner (OR: 1.6, 95% CI 1.1-2.3; p=0.007). It is reduced if enough breaks are taken (OR: 0.2, 95% CI 0.06-0.7; p=0.01).

**Conclusion:**

spinal MSDs were highly prevalent among dental professionals. Neck, back, and low back pain were significantly associated with occupational factors such as insufficient breaks, prolonged working hours, and lack of task rotation. These findings highlight the importance of ergonomic and organizational interventions in dental practice.

## Introduction

Musculoskeletal disorders (MSDs) include a range of inflammatory and/or degenerative conditions affecting the periarticular soft tissues of the limbs and spine [1]. They represent a major public health and occupational health concern, being strongly associated with a significant decline in quality of life and substantial socioeconomic costs [2]. A variety of sociodemographic, occupational, and psycho-organizational factors contribute to their onset [3,4]. In the hospital setting, spinal MSDs are among the most frequently reported health problems among healthcare professionals [5]. Dentistry is no exception, due to significant biomechanical constraints such as prolonged static postures, awkward positioning, exposure to vibrations, and the use of hand instruments requiring high manual precision [6]. According to a meta-analysis covering 41 studies conducted in Western countries, the prevalence of musculoskeletal pain among dental professionals’ ranges from 64% to 93%, with the most affected areas being the neck and lower back [3].

In Germany, the figures are even more striking: in a sample of 450 dentists and dental students, the 12-month prevalence of MSDs reached 92%, with the neck (78%) and lower back (58.7%) being the most commonly affected regions [7]. These spinal MSDs, particularly low back pain and neck pain, have major repercussions on both the professional and personal lives of dentists. They can lead to reduced productivity, functional limitations during clinical procedures, and a decreased ability to maintain prolonged postures. In the long term, chronic pain may result in repeated sick leaves, early career changes, or even premature retirement in severe cases [8]. The high prevalence of MSDs in dental practice fully justifies the need for targeted national and international studies. Such research not only helps quantify the extent of the problem locally but also identifies specific organizational, biomechanical, ergonomic, and psychosocial risk factors. This information is essential for developing ergonomic prevention strategies, improving dentists´ quality of work life, and reducing the socioeconomic burden associated with work absences or premature departure from the profession. In this context, this study aimed to identify the sociodemographic, professional, ergonomic and psychosocial factors associated with MSD of the spine and their prevalence among dentists at the Odontology University Hospital of Monastir and to propose a preventive approach based on work ergonomics.

## Methods

**Study design and setting:** this was an exhaustive cross-sectional analytical study conducted over a two-month period (from November 2018 to December 2018) among dental practitioners at the University Hospital Dental Clinic of Monastir, a reference institution in Tunisia for dental care, clinical training, and research in dentistry. This facility hosts both dental students in training and experienced practitioners, performing various clinical procedures. This setting provides an ideal environment for evaluating the impact of ergonomic and occupational constraints on the occurrence of spinal MSDs, due to the high postural demands, intense work pace, and prolonged exposure to repetitive technical tasks.

**Study design and setting:** this was an exhaustive cross-sectional analytical study conducted over a two-month period (from November 2018 to December 2018) among dental practitioners at the University Hospital Dental Clinic of Monastir, a reference institution in Tunisia for dental care, clinical training, and research in dentistry. This facility hosts both dental students in training and experienced practitioners, performing various clinical procedures. This setting provides an ideal environment for evaluating the impact of ergonomic and occupational constraints on the occurrence of spinal MSDs, due to the high postural demands, intense work pace, and prolonged exposure to repetitive technical tasks.

**Data collection:** data collection was carried out by a single investigator, who distributed the questionnaire to participants across the various departments of the Monastir Dental Clinic during break sessions. The self-administered questionnaire was in French and provided in paper format. Anonymity and confidentiality were strictly maintained. Sociodemographic data included gender, age, marital status, lifestyle factors (physical activity, smoking), and body mass index (BMI). Professional data collected included job status, department type (purely medical practice vs. medical-surgical practice), years of dental practice, daily working hours, number of patients, and number of practitioners per dental chair. Ergonomic characteristics of the workstation included working posture (sitting or standing), type of vision (direct or indirect), patient positioning (semi-seated or lying), presence of a dental assistant, and ergonomic aspects of the workspace (chairs, tools, and working area). The Nordic Musculoskeletal Questionnaire (NMQ) is one of the standardized tools used by occupational health physicians to screen for MSDs. It records the presence, intensity, and frequency of joint pain over the past six months of activity. In this study, we focused on spinal joint pain over the past 6 months. The results of the Nordic questionnaire were analyzed according to the location of the joint pain, particularly spinal regions: neck, upper back, and lower back [9]. A total score was calculated based on the number of affected joints. Participants were classified into two groups: No MSDs: No reported discomfort, pain, or issues in the past 6 months, Presence of MSDs: One or more affected joints. Psychosocial factors were assessed using the Karasek Job Content Questionnaire (JCQ), a widely used tool in epidemiological studies for predicting psychosocial and organizational work-related risk factors. We used the French version consisting of 26 questions covering three dimensions: psychological demand, decision latitude, and social support. Each item was scored using a 4-point Likert scale ranging from 1 ("strongly disagree") to 4 ("strongly agree"). Job strain in Karasek´s model is defined as high job demands combined with low decision latitude. Iso-strain combines job strain with low social support [10].

**Variable definition:** body mass index (kg/m^2^) was defined according to the World Health Organization (WHO) classification [11]: underweight: 15-19.9; normal weight: 20-24.9; overweight: 25-29.9; obesity class I: 30-34.9; obesity class II: 35-39.9; obesity class III: ≥40; professional status was categorized into two groups: trainees (interns and residents), seniors (assistant professors, associate professors, and professors); departments were grouped as follows: purely medical departments (dentofacial orthopedics, fixed prosthodontics, removable partial prosthodontics, complete prosthodontics, and conservative dentistry) and medical-surgical departments (oral medicine and surgery, periodontology, pediatric dentistry).

**Statistical analysis:** data entry was performed using the Statistical Package for the Social Sciences (SPSS), version 21. The dependent variable was the presence or absence of spinal MSDs. Statistical analysis was conducted in two stages: descriptive analysis: for quantitative variables, we calculated means, standard deviations, and medians, for qualitative variables, results were presented as frequencies and percentages. Analytical analysis: for comparing means of nominal variables, Student´s t-test was used, for comparing categorical variables, the Chi-square test was applied. Multivariable analysis was conducted using binary logistic regression. In univariable analysis, the significance threshold was set at p < 0.05. All variables with p < 0.20 were included in the multivariable regression model.

**Ethical considerations:** all personal data collected during this study were treated with strict confidentiality. Participant information remained anonymous to protect privacy. Ethical principles of research were rigorously followed in accordance with current recommendations. Informed consent was obtained from all participants. The study was approved by the Ethics Committee of the Faculty of Medicine of Monastir (CEFMM) and conducted in accordance with the Food and Drug Administration (FDA) and Office for Human Research Protections (OHRP) under the following registration numbers: Institution/Organization number: IORG0009738; CEFMM registration number: IRB00011572.

## Results

**General characteristics of the study population:** the total number of individuals in our study population was 260, with an overall participation rate of 64.23%, across all professional categories. Regarding sociodemographic characteristics, the typical profile of participants was: female (sex ratio = 0.41), right-handed (90.4%), with a mean age of 28.30 ± 6.7 years, single in 75.4% of cases, non-smoker (73.7%), practicing regular physical activity (56.1%), and with a mean body mass index (BMI) of 23.18 ± 3.39 kg/m^2^. Additionally, 79.6% of participants reported having no dependents. As for professional characteristics, 83% of participants were dental trainees (interns: 54.5%; residents: 29.09%). The majority (58.7%) worked in departments focused solely on medical practice. The median duration of dental practice was 12 months, while the average number of working hours per day was 5 ± 1.16 hours. With respect to workstation characteristics, 16.2% of participants reported never using a dental assistant during procedures. A total of 42.5% alternated between sitting and standing postures during clinical work. 57.6% performed procedures on patients positioned in a semi-seated posture. Half of the participants stated they did not take breaks between patients, and 92.8% reported not performing stretching exercises. A detailed description of the ergonomic characteristics of the workstation is presented in Table 1.

**Table 1:** general characteristics of the study population

Distribution of the study population according to the socio-demographic and professional characteristics		
Characteristics	Size	Percentage (%)
Gender		
Men	49	29.3
Women	118	70.7
**Marital status**		
Married	40	24
Single	127	76
**Smoking**		
No	123	73.7
Yes	42	25.1
**Regular physical activity**		
No	71	42.8
Yes	93	56.1
**Occupational status**		
Learner	140	83.8
Senior	27	16.2
**Practice service**		
For medical purpose	98	58.7
For surgical purpose	69	41.3
**Seniority (median)**	**12 months**	
	**Average**	**Standard deviation**
**Number of daily working hours**	5	± 1.16 hours
BMI (Kg/m^2^)	23.18	±3.39
Age	28.30	28.3
**Distribution of the study population according to the Ergonomic characteristics of the workstations**		
	**Size**	**%**
**Presence of a dental assistant**		
No	27	16.2
Yes	140	83.8
**Alternating standing and sitting positions**		
No	95	56.8
Yes	71	42.8
**Direct view**		
No	43	25.7
Yes	124	74.3
**Semi-sitting position**		
No	70	41.9
Yes	95	57.6
**Inter-patient breaks**		
No	94	56.3
Yes	73	43.7
**Stretching exercises**		
No	155	92.8
Yes	11	6.6
**Ergonomic chair**		
No	121	72.5
Yes	45	26.9
**Workspace**		
Suitable	57	43.3
Unsuitable	109	65.7

**Determinants of the onset of spinal musculoskeletal disorders (MSDs) among dental practitioners:** over the past 6 months, the prevalence of spinal MSDs in any site was 67.7% (113 cases). The involvement was multiple (≥2 concomitant locations) in 38% of cases. Upper back pain was reported by 43.7% of participants, followed by low back pain (33.5%) and neck pain (26.9%). Results of the univariable analysis showed that gender was significantly associated with both cervical pain (p=0.02) and back pain (p=0.009). Back pain was statistically associated with height (p=0.009) and regular sports activity (p=0.02). The main occupational and ergonomic factors associated with the different locations of back pain are shown in Table 2 with details. The concomitant presence of the upper limbs MSDs and the number of joints affected at the upper limbs were significantly associated with back pain (p=0.03 and p=0.003 respectively). The binary multivariable logistic regression model for the presence of neck musculoskeletal disorders (MSDs) included the female gender (aOR: 0.4, 95% CI: 0.18-0.9; p= 0.04), absence of breaks between patients (aOR: 0.2, 95% CI: 0.1-0.58; p= 0.002), presence of dorsal back pain (aOR: 3.8, 95% CI: 1.6-8.8; p = 0.001), and presence of low back pain (aOR: 6.2, 95% CI: 2.7-14.5; p= 0.001). Regarding factors associated with dorsal spinal MSDs, results showed that a low number of practitioners working on the same dental chair (aOR: 0.7, 95% CI: 0.66-0.95; p= 0.01), presence of upper limb MSDs (aOR: 1.43, 95% CI: 1.1-1.8; p= 0.007), absence of breaks between patients (aOR: 2.5, 95% CI: 1.2-5.3; p= 0.01), and associated neck pain (aOR: 3.3, 95% CI: 1.4-7.5; p= 0.004) increase the risk of dorsal back pain among dentists. The explanatory model for the occurrence of low back pain included lack of breaks (aOR: 0.2, 95% CI: 0.06-0.7; p= 0.01), presence of neck pain (aOR: 2.88, 95% CI: 1.3-6.2; p= 0.007), long working hours (aOR: 4.9, 95% CI: 2.2-11.1; p= 0.001), and trainee status in dental medicine (aOR: 1.6, 95% CI: 1.1-2.3; p= 0.007). Table 3 summarizes in detail the different factors associated with the onset of spinal MSDs by location after multivariable analysis.

**Table 2:** factors associated with spinal musculoskeletal disorders (MSDs) by location

Determinants of the onset of Neck pain	N		P
	MSDs+	MSDs-	
**Gender**			
Women	26	92	0.02
Men	19	30	
**Occupational status**			0.01
Senior	2	24	
Learners	43	98	
Break session: No	31	63	0.04
**Working posture**			
**Sitting**	16	30	0.15
**Standing**	29	92	
Sufficient workspace: No	26	84	0.18
Low back pain (+)	26	30	10-3
Upper back pain (+)	28	45	0.005
	**Mean ±SD**		**P**
	**MSDs+**	**MSDs-**	
Number of patients/day	3.3±1.9	4.1±4.1	0.1
Number of upper limb joints affected	4.8±1.5	4.41±1.4	0.09
**Determinants of the onset of upper back** pain	**N**		**P**
	**MSDS+**	**MSDS-**	
Gender			
Women	44	74	0.009
Men	29	20	
**Physical activity**			0.02
Active	49	47	
Inactive	24	47	
Break session: No	35	59	0.05
Sufficient workspace: No	44	66	0.17
Neck pain (+)	28	17	0.003
MSD-Upper limbs (+)	73	88	0.03
Determinants of the onset of upper back pain back	**Mean ±**SD		**P**
	**MSDs+**	**MSDs-**	
Height in meters	1.7±0.08	1.6±0.18	0.009
Number of practitioners per dental chair	2.8±1.7	23.2±3.4	0.01
Number of affected joints in the upper limbs	4.9±1.2	4.2±1.5	0.003
**Determinants of the onset of low back painback pain**	**N**		**P**
	**MSDs+**	**MSDs-**	
**Physical activity**			0.11
Active	37	59	
Inactive	19	52	
**Number of dependents**			0.16
0	48	85	
>0	8	26	
**Occupational status**			0.04
Senior	4	22	
Learners	52	89	
Break session: No	27	67	0.13
Ergonomic chair: No	37	85	0.14
Neck pain (+)	26	19	10-3
MSD-Upper limbs (+)	56	105	0.18
Number of daily working hours	5.2±1.3	4.9±1	0.14
Number of affected joints in the upper limbs	4.8±1.1	4.3±1.6	0.013

**Table 3:** univariable and multivariable binary logistic regression model explaining the occurrence of spinal MSDs by location

Occurrence of neck pain	Univariable			Multivariable binary logistic regression model	
Variables	Number		P-value	OR (95%CI)	P-value
	MSDs+	MSDs-			
**Female gender**	26	92	0.02	0.4 (0.18-0.96)	0.04
**Lack of break sessions**	31	63	0.04	0.2 (0.1-0.58)	0.002
**Upper back pain (+)**	28	45	0.005	3.8 (1.6-8.8)	0.001
**Low back pain (+)**	26	30	10-3	6.2 (2.7-14.5)	10-3
**Occurrence of upper back pain**	**Univariable**			**Multivariable binary logistic regression model**	
	**MSDs+**	**MSDs-**	**P-value**	**OR (95%CI)**	**P-value**
Lack of break sessions	35	59	0.05	2.5 (1.2-5.3)	0.01
Neck pain	28	17	0.003	3.3 (1.4-7.5)	0.004
**Insufficient number of practitioners per dental chair**	2.8±1.7	23.2±3.4	0.01	0.7 (0.66-0.95)	0.01
**Number of upper limb disorders**	4.9±1.2	4.2±1.5	0.003	1.43 (1.1-1.8)	0.007
**Occurrence of low back pain**	**Univariable**			**Multivariable binary logistic regression model**	
	**MSDs+**	**MSDs-**	**P-value**	**OR (95%CI)**	**P-value**
**Lack of break sessions**	27	67	0.13	0.2 (0.06-0.7)	0.01
**Neck pain (+)**	26	19	10-3	2.88 (1.3-6.2)	0.007
**High number of daily working hours**	5.2±1.3	4.9±1	0.14	4.9 (2.2-11.1)	10-3
**Occupational status: trainees**	52	89	0.04	1.6 (1.1-2.3)	0.007

## Discussion

The primary objective of our study was to determine the prevalence of musculoskeletal disorders (MSDs) affecting the spine, particularly cervicalgia, dorsalgia, and low back pain, among dentists working at the University Dental Hospital of Monastir. These conditions are especially common in this population due to the specific postural demands of dental practice, including prolonged static positions, repetitive movements, and a frequently non-ergonomic work environment. In parallel, the study aimed to identify sociodemographic, occupational, and psychosocial factors associated with the occurrence of these spinal MSDs. The ultimate goal was to highlight potentially modifiable determinants of these disorders in order to propose appropriate preventive measures that could improve quality of work life and promote the sustainability of careers in oral healthcare. Among the 260 eligible practitioners, the participation rate was 64.23%. The majority of participants were female (70.7%), right-handed (90.4%), with a mean age of 28.3 ± 6.7 years, 83% of the participants were trainees. The median duration of professional experience was 12 months, while the average number of daily working hours was 5 ± 1.16 hours. In the present study, the prevalence was 67.7%, with 41.7% of cases being mono-localized. According to the literature, the prevalence of spinal MSDs ranges from 60% to 90% among dentists [12].

The high frequency of these conditions is attributed to microtrauma resulting from tendinous and muscular overuse, prolonged maintenance of constrained postures, and psychosocial stressors [13,14]. Contrary to our findings, the literature generally reports neck pain as the most common site of spinal MSDs [15]. In our study, female gender, lack of scheduled breaks, and the presence of dorsolumbar pain were significantly associated with cervical pain. As for upper back pain, we observed a prevalence of 43.7%, which was associated with the absence of breaks, limited rotation of practitioners at the dental chair, the presence of cervical pain, and concurrent upper limb MSDs. This figure is consistent with previously reported prevalence rates in the literature, which range from 40% to 53%. Several authors attribute back pain to paravertebral muscle fatigue due to sustained and awkward postures during patient care [16]. Low back pain accounted for 33.7% of all spinal disorders, falling within the prevalence range reported in the literature (22% to 69.8%) [17]. Its occurrence in our study was associated with trainee status, concomitant neck pain, absence of break periods, and a high daily workload.

Our study demonstrated, in line with findings from the literature, that trainee status was a significant risk factor for both neck pain and low back pain. Actually, the reported prevalence of cervical pain varied from 83.4% to 85.7% and was significantly higher in young dentists at the beginning of their career [18]. This may be attributed to the higher number of patients treated by learners compared to seniors. According to Huang *et al*. this is explained by the lack of experience and knowledge of MSDs in these learners and limited clinical experience and a higher exposure to stressful or physically demanding tasks during the early stages of professional practice [19]. Organizationally, the large daily workload increases the risk of low back pain by a factor of 4.9 for the dentists in our study. In the literature, long work sessions lead to cervical and low back pains due to the large number of patients, which requires a fixed work posture, repetitive and continuous movements and enormous physical and emotional stress [14]. In our study, the absence of break periods increased the risk of back pain (aOR: 2.5, 95% CI: 1.2-5.3; p=0.01). Conversely, when breaks were taken regularly and sufficiently, the risk of both neck pain and low back pain was reduced respectively (aOR: 0.2, 95% CI: 0.1-0.58; p=0.002), (aOR: 0.2, 95% CI: 0.06-0.7; p= 0.01). Prolonged maintenance of a static posture without rest leads to continuous contraction of the cervical paravertebral muscles, necessary to support the head in a forward position. This results in the loss of cervical lordosis and increases the risk of cervical disc herniation [19].

The coexistence of spinal MSDs at different levels was observed among our practitioners, consistent with findings reported in [20]. There is a close relationship between the neuromuscular stability of the thoracic and lumbar spine and the function of the cervical spine. A sustained faulty dorsal posture can lead to the development of cervical pain. Indeed, maintaining proper spinal alignment requires continuous adjustments of the cervical and lumbar curves, which generates significant muscular tension in the back muscles. This reflects a compensatory mechanism in which a tilt or rotation at one spinal segment is offset by adjustments in other segments to preserve overall spinal stability [21]. The most effective preventive approach to spinal (MSDs) should rely on a comprehensive and participatory strategy addressing the various contributing factors, in order to develop cost-effective, feasible, and applicable interventions. Introducing a culture of ergonomic working postures from the early stages of dental education is essential [22], particularly through hands-on practical sessions and self-confrontation techniques [23]. Studies have reported a reduction in MSDs-related absenteeism rates following such interventions [24]. In terms of workspace design, incorporating a mobile unit or a telescopic arm can enhance the accessibility of instruments. The dentist´s chair should be equipped with adjustable lumbar support and casters to facilitate movement. In cases of back problems, a saddle stool or a sit-stand chair may help maintain a more natural lumbar curve.

Repositioning the patient´s head during treatment also helps reduce spinal flexion and improves access to various areas of the oral cavity. Alternating between sitting and standing postures throughout the day is highly recommended [25]. Implementing regular micro-breaks is also strongly encouraged, as these provide dentists with the opportunity to stretch and allow patients to relax their jaw muscles [26,27]. Efficient workload management is key: dividing lengthy procedures into multiple sessions, alternating complex and simple cases, and avoiding extended workdays without adequate rest are all important strategies [25]. Furthermore, stress management should be integrated into the dental curriculum [28]. Promoting self-esteem, managing expectations, and enhancing job satisfaction are crucial for improving mental well-being. Encouraging team-building initiatives within clinical departments can further strengthen peer support and workplace recognition, contributing to better team cohesion [29]. This study is the first to comprehensively investigate MSDs of the spine among dental practitioners at the Monastir dental clinic in Tunisia, addressing a key gap in regional occupational health research within this region. By including the entire clinic staff, it provides a broad view of MSD of spine prevalence and potential risk factors. The self-administered questionnaire allowed for quick, cost-effective data collection and likely encouraged honest responses due to its anonymity. However, some items in the questionnaire could have been interpreted differently according to the participants and considered too long, thus encouraging participants to ignore them. Other sources of bias were raised: at the end of the shift, some left their work early and could not be integrated into the study, the constraint of time or, sometimes, the presence of the interviewer could influence the answers.

## Conclusion

At the end of this study, it appears that dentistry is a profession at high risk of spinal MSDs due to the omnipresence of multiple biomechanical and psycho-organizational constraints within this discipline. The preventive approach to these disorders in dentistry should focus primarily on the detection of high-risk work situations with an ergonomic study of the various workstations in order to improve the work equipment. Ergonomics education should be introduced early to familiarize trainees with the basic principles of space, tool and gesture ergonomics at the start of their training. Regular micro-breaks are highly recommended and should be introduced at the beginning of training. Early detection of signs of spinal MSDs in dentists is the responsibility of the occupational physician, and must be carried out regularly, in order to make the necessary adjustments or even occupational redeployment before leaving the profession and early retirement due to premature physical decline or disability

### What is known about this topic

- Spinal musculoskeletal disorders are a significant health problem with major economic and psychosocial impacts;
- Dentists are highly exposed to spinal musculoskeletal disorders due to biomechanical, psychosocial, and organizational factors;
- These disorders can cause irreversible physical damage, disability, and early retirement.

### What this study adds

- Raise awareness among dentists of the spinal musculoskeletal disorders risk factors associated with their profession;
- Promote simple and effective prevention to improve quality of life at work;
- Integrate ergonomic education early to instill good habits in posture, workspace, and tool use.
